# Oxygen in Its Relationship to the Vital Phenomena

**Published:** 1871-09

**Authors:** J. F. Sanborn

**Affiliations:** Tabor, Iowa


DENTAL REGISTER.

VOL. XXV.] SEPTEMBER, 1871. [NO. 9.

### Communications.

### OXYGEN IN ITS RELATIONSHIP TO THE VITAL PHENOMENA.

BY J. F. SANBORN, M. D., TABOR, IOWA.

An Essay read before the Iowa State Dental Society, held at Council Bluffs, July 13th, 1871.

*Mr. President, and Members of the Dental Profession:*

It is with pleasure I again meet you at this, our annual gathering, and again invite your attention to an Essay upon a subject strictly physiological.

It is very natural for us to study effect, rather than the cause, and the relationship existing between cause and effect.

If, as shown by our Essay last year, life is the sum of all the vital changes, then the study of the relationship of these changes is a subject worthy of the profoundest philosopher.

The first act in all animal existence is the breathing of atmospheric air, the vital principle of which is oxygen; the suspension of breathing is followed by death; therefore, in order to enjoy life and health, air must be supplied regularly, in full quantity, in right proportions, and uncontaminated with foreign gases.

Pure atmospheric air is composed of four parts of nitrogen to one of oxygen. This proportion is maintained in all parts of the inhabitable earth; to the dwellers on the higher elevations as well as the lowest parts of the earth's surface, in the wilds of the jungle, or on the rolling ocean chemical analysis has never been able to detect any difference.

Oxygen was first discovered by Dr. Priestly, in 1774, and was by him called "vital air," but was named oxygen by Lavoisier because at that time it was supposed to be the active principle in all acids: the discoveries of modern chemistry have demonstrated this to be an error.

Oxygen is a gas, colorless, tasteless, and inodorus; we can neither see or feel it, yet it enters into the composition of the largest portion of our globe.

In nature it is never found pure but is always chemically, or mechanically combined with some other substance. Its weight is one tenth more than atmospheric air. Water is a combination by weight of oxygen, 8 parts to 1 part of hydrogen; in bulk, oxygen 1 part, to hydrogen 2 parts. Hydrogen is the lightest of all known substances, and is 16 times lighter than oxygen.

Oxygen is the most widely diffused of all the elements; it constitutes one-fifth of the atmosphere, eight-ninths of the water, and nearly one-half of every solid substance we see around us.

Oxygen is supposed to exist both in a quiescent and active state: the latter is known as ozone.

As our subject is oxygen rather than ozone, we leave the latter for the investigation of others.

There is a distinction to be made between chemical and vital action, the latter may not be in violation of chemical law, but it is of a higher class of actions, and is the development of a higher state of matter from that of a lower, while chemical action is disintegrating or breaking down of organic matter, and restoring it to the quiescent, inactive, inorganic, non-vital condition.

We find oxygen associated with nitrogen in the following proportions:

Nitrogen four parts, oxygen one part, to constitute common air.

N. 1, O. 1—Nitrous oxide.

N. 1, O. 2—Deut. oxide of nitrogen.

N. 1, O. 3—Hypo nitrous acid.

N. 1, O. 4—Nitrous acid.

N. 1, O. 5—Nitric acid.

It will be seen that nitrogen and oxygen have uncommon affinity for each other; there being six different combinations, five of which are chemical; and with each additional proportion of oxygen to one of nitrogen, is the compound ready to part with one or more of its proportions of oxygen to any other substance; this is carried to such a degree of activity as to pass from pure benevolence to aggressive warfare, and so forces its super-abundance of oxygen on other substances, and disintegrates them.

This may be seen by the readiness with which nitric acid destroys whatever it comes in contact with.

Atmospheric air is but a mechanical mixture, so the oxygen can be readily separated from the nitrogen to support animal life.

The nitrous oxide is equal parts of oxygen and nitrogen chemically combined, and much used for anæsthesia, notwithstanding the two inseparable obstacles to its being a true "vital air."

1st. It is a chemical combination of the two gases.

2d. The unphysiological proportions. And as has been stated, atmospheric air is oxygen, one part to four of nitrogen. Nitrous oxide is one of each.

Nitrous oxide, chloroform, or any other inhaling anæsthetic do but deprive the vital economy of its natural supply of oxygen.

In order to the full, free and well balanced vital action to constitute respiration, three things are necessary.

1st. The natural supply of air to furnish the blood with oxygen so essential, that a deprivation of it causes death in a few minutes, and is known as death by asphyxia.

2. The circulation is in part the conveyance of the oxygen to where it is wanted.

3d. The brain power that regulates these actions, is itself dependent on the other two actions to keep itself in full, free action.

The inhalation of an anæsthetic in place of air, deprives the system of oxygen, in part at least, which destroys the balance. The cerebrum, the seat of sensibility, is supplied with blood charged with a poisonous drug, in place of its accustomed oxygen, which paralyzes it, and you have the anæsthesia desired, but it is occasioned by an unphysiological relationship between the brain and what? 1st. Negatively—The brain is deprived of oxygen. 2d. The blood is charged with carbonic acid gas and other broken down matter, that should and would be removed under the ordinary action of normal breathing, but are retained for want of oxygen to remove them, and by their presence poison the whole system, and especially derange the functions of the brain; thus anæsthesia is produced by paralysis.

3d. The brain may be influenced by both these unphysiological relationships, and the sedative action of the drug. The fumes of burning charcoal in a close room, are but common air deprived of oxygen and carbonic acid gas, or nitrogen and carbonic acid gas; both incapable of supporting life, yet if any person should breathe for any length of time such a combination of gases, anæsthesia would be produced and death would soon follow, not from the azotic condition of the gases, but from the asphyxia or depression of the system for want of oxygen.

In giving an anæsthetic there is undoubtedly sometimes a closing of the epliglottis, and death by asphyxia is the result.

The paralysis of the brain from whatever cause may and does sometimes extend from the cerebrum to the cerebellum, and the lamp of life is puffed out in an instant, and the friends are called upon to mourn the wonderful dispensation of Providence.

From like cause the numo-gastric nerve may become paralyzed and the lungs cease to act; or the sympathetic nerve may become paralyzed, and the heart cease to act and death be the result.

How appropriate are the words of Prof. O. W. Holmes:

> "God gave his creatures light, air,
> And water open to the skies,
> Man locks him to a stifling lair
> And wonders why his brother dies."

These are my objections to anæsthetics.

Physiology is not an exact science, like mathematics. There is a diversity of opinions entertained by various authors in regard to the use oxygen subserves in the animal economy.

Your attention is invited to some of these, together with our conclusions, all of which you will weigh in the balance of your good judgment, and "govern yourselves accordingly."

Dr. A. E. Ames, of Minneapolis, Minn., in a conversation a few weeks since, on the source of animal heat, says substantially as follows: "By the oxy-hydrogen blow pipe the greatest degree of artificial heat is obtained, and that without light, but silently the most refractory of the metals are fused. In like manner the oxygen received into the lungs unite with the hydrogen everywhere present in the system; by their union water is formed and animal heat produced.

The evaporation of the water regulates the temperature of the body, and thus is beauty and harmony preserved."

Lavoisier believed oxygen received in the lungs was there combined with the carbon in the blood, and immediately exhaled as carbonic acid gas; the lungs really acting the part of a stove to burn the carbon, and in this way animal heat is produced. Liebig's theory is the same.

If this were the true theory the lungs would be the warmest part of the body. The fact is they are the coldest.

Carpenter thinks the oxygen is carried to every part of the body in the arterial blood, and combines with the hydrocarbon in the capillary system to produce animal heat.

Dalton says, "with regard to the mode of generating of the internal or vital heat, we start with the assertion that its production depends upon changes of a chemical nature; and is so far to be regarded as a chemical phenomena."

"The numerous combinations and decompositions which follow each other incessantly during the nutritive process, results in the production of internal or vital heat."

So much for the chemical theories.

Prof. Payne, of New York University Medical College says, "Neither the union of the oxygen of the air with the carbon of the blood or with that of the body in the act of respiration, or excretion of carbonic acid gas has any greater connection with the production of animal heat than with the gastric juice, or any other result of organic formation.

Bicart says, "The state of the respiration has no influence on the actual heat of the body."

Prof. Payne says, "When we place on one side all the phenomena of animal heat, and on the other the chemical hypothesis, it appears to me so inadequate to the explanation, that I think every methodical mind can refute it without any assistance."

John Hunter says, "The power to generate animal heat in animals, arises from a principle so connected with life that it can, and does act independent of the circulation, and is that power which preserves and regulates the internal machinery."

Muller says, "There must be still some other source of animal heat than respiration."

He places it in the organic resources in which by the organizing forces on the organic matter, heat is generated; not in one, but in every organ of the body.

Treadman, holds to the same views.

Who shall decide when such Doctors disagree?

That the inhalation of atmospheric air; the absorption of oxygen by the blood, and the exhalation of carbonic acid gas are essential to vitality, all are agreed; but where the change takes place, and the nature of the process, there is quite a diversity of opinion.

I have been unable to find in what consists the vital action, or the modus operandi of the production of animal heat as believed by the vital theorists.

In default of others to render a satisfactory explanation, I propose to place myself on the record as solving the mystery, and here present a theory that I think fills the demands of the case.

The use of oxygen in the vital economy, is so intimately connected with animal heat, that it is quite out of the question to discuss one without having the other somewhat in view.

The sun is the source of light and heat to our globe.

The rays of the sun heat some places more than others, this difference in temperature causes the winds to restore the equilibrium.

The winds and the sun in evaporating moisture, the cold currents of air coming in contact with the warm ones saturated with moisture, causes its condensation, and it drops in rain.

The warmth of the sun and the moisture of the rain, causes vegetation to grow.

The gentle breezes and high winds give a variety of motion, which is the exercise of vegetable life; which causes it to strike its roots deeper into the soil where a greater supply of nourishment is furnished, and a greater growth obtained.

Surely there is harmony in the operations of nature.

In this growth there is an expenditure of solar heat, which is stored up in the vegetable tissue, be it wood, fruit, grass or seed; wherever there is growth, there is an accumulation of heat, which is carefully stored up for the use not of itself, but for man's comfort and convenience.

The coal taken from far below the earth's surface to supply the length and breadth of the land with fuel; and the petroleum that casts so much light on the operations and designs of man, and adds so much to his comfort and pleasures, are but the reserved heat of the sun accumulated long ages, before Adam's creation.

The heat of the sun is apparent heat; that stored up in vegetation is latent heat, which will there remain thus stored up, independent of the surrounding temperature, however long continued.

We might have thousands of cords of wood seasoned ready for the fire, and yet perish for want of the latent heat therein contained.

Thus we can obtain by elevating the temperature of the wood until oxygen will readily combine with its structure and reduce it to the original elements of which it is composed.

The unconsumed vapors, and carbonic acid gas pass away into the great ocean of atmosphere, the chaos from which it was derived, and the remainder of the wood is reduced to ashes.

The organic wood has by the agency of oxygen been reduced to its original elements, and in this disintegration, or breaking down of wood structure, the latent heat stored up years before, has become apparent heat, and we are permitted to enjoy its benign influence.

The greater the avidity with which oxygen combines with any substance, the quicker it is disintegrated and the greater is the amount of apparent heat we are permitted to enjoy. Had the wood undergone the slow process of decay, precisely the same amount of heat would have been liberated and have been imparted to the surrounding atmosphere, even though it should have been hundreds of years in decaying.

Heat and motion are interchangeable conditions, but like matter are never annihilated.

Vegetation is the reservoir for the storing up of apparent heat so abundantly supplied during a goodly part of the year.

The grass contains it; so does the oats and corn, the wheat and all classes of food.

The horse and ox build up their structure from the grass, hay and grain.

The food for the animal kingdom must be the result of growth, either vegetable, or animal, in order to contain proper material to form tissue, which must be digested and becomes nutritious matter in the blood, which must be carried to every part of the system, wherever it is wanted, it is used to build up and supply the waste tissues.

We are continually breathing oxygen from the surrounding air, this is conveyed to the red corpuscles of the blood in every part of the body, and is there ready provided, wherever and whenever wanted to unite with the tissues, disintegrating them or reducing them to a broken down condition, just as the wood is reduced to ashes, and in this breaking down of tissue, the latent heat therein contained becomes apparent and the real source of animal heat.

Wherever this disintegration takes place, there the carbonic acid gas is found, and is immediately passed to the lungs and liver for elimination.

It will be seen that we make oxygen a universal breaking down agent, and not building up agent.

The decay of all organic matter is by its action; all combustion is maintained by its presence, even the solid rock is not proof against its influence; the iron and steel are corroded by it and reduced to a worthless substance; even gold and platinum are not proof against its influence.

Life is action, the sum of the vital changes, the tear down is as much a vital process as the build up, and oxygen is a "vital air," performing its essential vital action not in building up, but in breaking down, and animal heat is one of the vital changes, the effect of which, the disintegrating action of oxygen on the vital tissues is the cause, and wherever the breaking down takes place, there is animal heat generated.

Physical exercise increases the breaking down of the tissue; this creates a demand for an increased supply of oxygen for that purpose and as a consequence, there is an increase of animal heat.

This in severe physical exercise is familiar to every one, and may be seen in animals exposed to long continued cold; the shivering is but involuntary exercise made to increase animal heat, and is explainable on the above principles.

From birth to death the first and most imperative demand is for air, the active principle of which is oxygen, and its introduction is the fundamental event of animal life; all other physical operations are subordinate to this, and depend upon it.

We eat, digest, and assimilate food, only that it may finally administer to the respiratory process, and be disintegrated by oxygen.

It destroys a part for the good of the whole, and is thus the great motor of animal vitality.

If we in any manner touch the relationship of oxygen to the living system, the vital machinery is at once deranged. The use of anæsthetics of whatever character, for the time being, disturbs this relationship, and the frequent deaths from their use, proves the truth of this assertion, and the danger of such a practice.

Nature's demand is true obedience to physiological law.

